# A mixed-methods observational study of strategies for success in implementation science: overcoming emergency departments hurdles

**DOI:** 10.1186/s12913-024-12102-9

**Published:** 2025-01-27

**Authors:** Deonni P. Stolldorf, Alan B. Storrow, Dandan Liu, Cathy A. Jenkins, Rachel A. Hilton, Karen F. Miller, Joy Kim, Deepika Boopathy, Satheesh Gunaga, Bory Kea, Joseph Miller, Sean P. Collins

**Affiliations:** 1 Vanderbilt University School of Nursing, Nashville, TN USA; 2 Emergency Medicine, Vanderbilt University Medical Center, Nashville, TN USA; 3 Vanderbilt University Medical Center, Nashville, TN USA; 4 Department of Biostatistics, Vanderbilt University Medical Center, Nashville, TN USA; 5 Department of Emergency Medicine, Center for Policy and Research in Emergency Medicine, Oregon Health & Science University, Portland, OR USA; 6 Department of Public Health Sciences, Henry Ford Health, Detroit, MI USA; 7 Department of Emergency Medicine, Henry Ford Wyandotte Hospital, Wyandotte, MI USA; 8 Emergency Medicine, Henry Ford Health and Michigan State University, Detroit, MI USA; 9 Emergency Medicine, Vanderbilt University Medical Center and, Veterans Affairs Tennessee Valley Healthcare System, Geriatric Research, Education and Clinical Center (GRECC), Nashville, TN USA

**Keywords:** Heart failure, Self-care, Implementation, Emergency department, Care transitions

## Abstract

**Background:**

Heart failure is a major public health concern, affecting 6.7 million Americans. An estimated 16% of emergency department (ED) patients with acute heart failure (AHF) are discharged home. Our Get with the Guidelines in Emergency Department Patients with Heart Failure (GUIDED-HF) toolkit aims to improve AHF self-care and facilitate safer transitions in care for these patients. We describe implementation barriers and facilitators, and the selection and refinement of implementation strategies, to facilitate future GUIDED-HF implementation.

**Methods:**

A mixed-methods cross-sectional observational study was conducted in four United States EDs in two diverse healthcare systems in the Pacific West and Midwest. Data were collected using a survey and interviews with ED providers, nurses, and leaders. The survey assessed the ED context using the context scale of the Organizational Readiness to Change Assessment (ORCA). The Consolidated Framework for Implementation Research informed interviews. Quantitative data were summarized using medians (interquartile ranges) or percentages (frequencies). Wilcoxon rank-sum tests and Kruskal–Wallis tests were used to assess differences in the healthcare system and profession. Qualitative data were analyzed and summarized using rapid qualitative analysis. Convergence of quantitative and qualitative data was used to inform specific refining of implementation strategies to the local context (e.g., who should serve as champions, how best practice alerts should be implemented).

**Results:**

Participants were predominately white (76%) with median (IQR) age 37.0 (32.0, 41.0). ED leaders/administrators, providers, and nurses comprised 15%, 55%, and 29% of participants, respectively. Sites reported an ORCA context scale score of 3.7 [3.4, 4.0] (scale of 1 = *strongly disagree* to 5 = *strongly agree*). Comparison of scores by profession showed a significant difference in the context score among providers (3.9 [3.5, 4.0]), leaders (3.7 [3.5, 4.0]), and nurses (3.6 [3.0, 3.9]) (*p* = 0.048). Qualitative data indicated implementation barriers (e.g., resource limitations, patient health literacy), facilitators (e.g., GUIDED-HF is patient-centric; site and intervention congruent values, norms, and goals), and site-specific needs due to contextual factors (e.g., education needs, feedback mechanisms, champions).

**Conclusions:**

Specific determinants of implementation exist in ED settings and require the refining of implementation strategies to overcome site-specific barriers and enhance facilitators.

**Trial registration:**

n/a.

**Supplementary Information:**

The online version contains supplementary material available at 10.1186/s12913-024-12102-9.

## Contributions to the literature

- GUIDED-HF is a self-care intervention for patients with acute heart failure discharged home from the emergency department (ED), addressing the paucity of interventions to improve their transition from ED to home.
- This study demonstrates the nuanced contextual differences between implementation sites and the need for a deliberately selected and refined implementation approach addressing the specific concerns and preferences of the local context.
- The findings contribute to existing knowledge gaps of best practices for implementing complex healthcare interventions in the fast-paced ED setting.

## Background

Effectively implementing interventions to improve outcomes in patients with heart failure (HF) remains an urgent need. Approximately 6.7 million US adults live with HF, and in the first 5 years following diagnosis, 83% are hospitalized at least once, and 54% are admitted three or more times [1]. These patients often experience worsening signs and symptoms, referred to as acute heart failure (AHF), requiring emergency care. Most ED patients are hospitalized, and over 18.2% and 31.2%, respectively, experience unplanned 30-day and 90-day HF readmission or mortality after hospital discharge [2]. Approximately 16% of patients with AHF are discharged home directly from the ED and of these 24% subsequently return to the ED or are admitted, necessitating the implementation of evidence-based interventions that improve ED-to-Home transitions [3, 4]. Self-management interventions in heart failure is a proven, acceptable method for enhancing ED-to-Home transitions, [3, 5, 6,7] including our GUIDED-HF self-care intervention, which improves ED-to-Home care transitions and reduce ED visits and hospital readmissions [6].

**Fig. 1:**
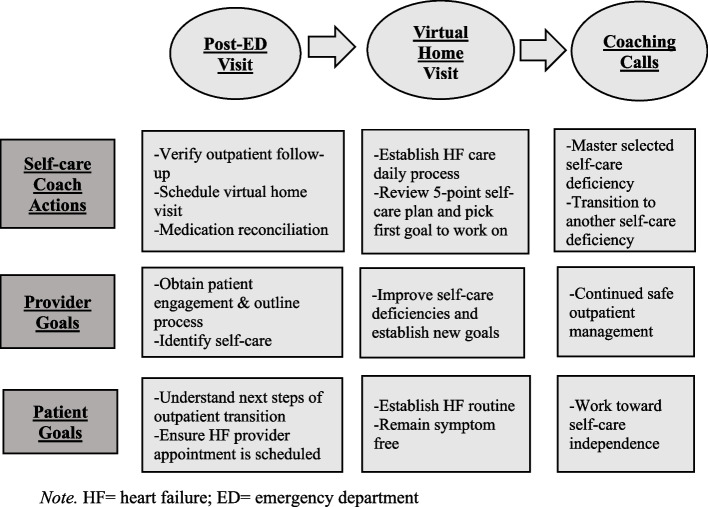
GUIDED-HF multifactorial care transitions intervention

GUIDED-HF was previously developed and informed by the Care Transitions Intervention framework (Fig. 1), [8–11] which consists of three intervention stages: 1) hospital/ED visit, 2) home visit, and 3) follow-up coaching calls. These stages focus on self-care coaches presenting an overview of GUIDED-HF during the post-discharge ED visit and facilitating patient mastery of self-care items during the home visit and coaching calls (Fig. 1). ED providers refer eligible patients to the self-care coaches, who then contact the patients by phone to discuss GUIDED-HF and gauge patient interest in receiving the intervention. Over a 1-month period, a home visit occurs, followed by two coaching calls. Our prior multi-center randomized controlled trial (NCT02519283)(2015–08–10) (Clinicaltrials.gov) [6] comparing GUIDED-HF with usual care in patients with AHF discharged from the ED, showed statistically and clinically significant improvements in a 30-day global rank of cardiovascular death, ED revisits or hospitalization for AHF, overall symptom burden, and HF knowledge [6, 12, 13, 14].

Implementing interventions like GUIDED-HF in the fast-paced, complex ED setting with high patient turnover, various patient acuity levels, and complex care delivery can be challenging. Implementation barriers in ED settings include insufficient resources, lack of sufficient training and education, competing priorities, and insufficient time for implementation. However, using implementation science, the scientific study of methods to enhance the uptake and sustainability of evidence-based interventions, [15, 16] can bring clarity to implementation determinants and how best to achieve fit between the implementation context and strategies used to implement an intervention. [15–18] Developing implementation strategies is a necessary step towards generating organizational change [16, 19–24]. Studies have also highlighted the need to assess how different implementation contexts dictate unique barriers and facilitators to outcomes such as reach, adoption, effectiveness, fidelity, and sustainability [19, 25, 26]. Thus, identifying contextual determinants that might impede or enhance (i.e., barriers and facilitators) the implementation of GUIDED-HF and selecting implementation strategies to address known barriers and facilitators was a crucial first step towards its effective implementation and subsequent clinical effectiveness. Matching implementation strategies with the finer nuanced contextual differences in the ED setting was important for long-term sustainment of GUIDED-HF.

We, therefore planned for a Hybrid Type II implementation-effectiveness study to translate GUIDED-HF into real-world settings in two geographically diverse health systems representing four high-volume EDs in the United States and evaluate the implementation outcomes of reach, effectiveness, adoption, implementation and maintenance (RE-AIM). For an implementation approach that was practical and feasible to ED settings, and to prepare for the hybrid type II implementation formative evaluation, we first used a mixed-methods approach to identify barriers and facilitators, and select and refine strategies for GUIDED-HF implementation. We used a mixed-methods approach to quantitively measure the implementation context followed by a qualitatively exploration of implementation strategies to address contextual barriers and facilitators.

Our objective was to report the ED-specific barriers and facilitators we identified and the nuanced differences between sites informing the selection, addition and/or adjustment of our a priori implementation strategies to guide the subsequent Hybrid Type II implementation formative evaluation [27–29].

## Methods

### Overview

In preparing our implementation plan (Table 1), we proposed and used our prior approach of a similarly complex interdisciplinary intervention [30–34], implemented in the ED and hospital. This was informed by extant literature reporting effective implementation strategies for use in ED settings, [17, 18] the research team’s experiences with implementation, [34] and the ERIC (Expert Recommendations for Implementing Change) taxonomy of implementation strategies [20]. However, we anticipated and remained open to adding strategies as needed to address barriers and facilitators we identify in the course of our work. Our goal was to refine these strategies based on survey and interview results to address fine nuanced contextual differences between EDs. For example, based on anecdotal information from site PIs, we anticipated differences in ED workflow processes to facilitate communication with self-care coaches and eligible GUIDED-HF patients after ED discharge. We also anticipated education materials and strategies might differ to accommodate differences in ED staffing patterns between facilities. Our work aligns with recent recommendations for the development of implementation strategies that address local circumstances in ED settings, [35] and the use of qualitative methods [36] and well-known implementation frameworks and taxonomies in implementation studies [16, 37]. Our work will also enable clinicians and implementers to evaluate their contextual determinants against what we describe in this report and the usefulness of our selected and refined implementation strategies in their settings to effectively implement similarly complex interventions.

**Table 1:** A priori implementation strategies with definitions

A. CATEGORY: Planning	
A.1. Develop stakeholder buy-in:	
-Use of champions	Prepare and train self-care coaches to serve as champions and provide them with resources to market/advocate for GUIDED-HF. Potential champions: principal investigators, department chairs, ED staff, and self-care coaches
-Consensus discussions	Include providers and other stakeholders, including self-care coaches, in discussions on the importance of GUIDED-HF and its continued use in the ED setting throughout the study. Potential targets: ED providers, self-care coaches, and opinion leaders at each site
A.2. Marketing	Use strategies that will increase the awareness and knowledge of staff on GUIDED-HF. Potential strategies: study flier for distribution by site champions
B. CATEGORY: Educate	
B.1. Conduct educational meetings	Hold meetings targeted toward providers, administrators, other organizational stakeholders, and community, patient/consumer, and family stakeholders to teach them about the clinical innovation. Develop tools and guidelines to support stakeholder learning
B.2. Develop and distribute educational material	Develop educational material for GUIDED-HF. Potential distribution strategies: email, print, word-of-mouth/in-person, workstation reminders
C. CATEGORY: Quality Management	
C.1. Adapting workflow processes	Change workflow processes to facilitate integration of the intervention. Potential strategies: create a checkbox for GUIDED-HF documentation or best practice alert
D. CATEGORY: Restructuring	
D.1. Audit and feedback	Collect and summarize clinical performance data over a specified time and give it to clinicians and administrators in the hopes of changing provider behavior

*ED* Emergency department

### Study design and setting

A mixed-methods convergent QUANT→QUAL cross-sectional observational study design was used in four EDs in two large United States (US) healthcare systems located in the Pacific West (HC-PW) and the Midwest (HC-MW). The sites are geographically and socio-demographically diverse, with HC-PW serving a large community of privately insured, mostly white patients and HC-MW serving a large urban population of uninsured and underinsured Black patients. This diversity of healthcare settings included tertiary, community and freestanding EDs and was ideal for developing a refined implementation approach for future dissemination across equally diverse US EDs. The sites’ self-care coaching personnel were also diverse. HC-PW used staff who previously served as research coordinators in the ED to serve as study self-care coaches. HC-MW integrated the delivery of GUIDED-HF in their mobile integrated health unit, with community paramedics serving as self-care coaches, allowing for rapid in-home follow-up after ED discharge.

### Participant characteristics

Participants were ED providers who were responsible for assessing HF patients, determining their diagnosis, and providing treatment (i.e., physicians, advanced practice nurses, and physician assistants) as well as ED nurses, and ED leaders/administrators.. We asked participants to self-identify to the group they affiliated with and if they identified with more than one group, to choose the group reflective of their primary role. Inclusion criteria were 1) current healthcare system employee, 2) aged ≥ 18 years, 3) one of the following: ED providers and nurses directly involved in patient care; ED managers, supervisors, educators, or clinical nurse specialists; or self-care coaches.

### Data collection

#### Surveys

Surveys were administered using REDCap® (Research Electronic Data Capture), a secure web-based platform for online surveys [38, 39]. Participants were recruited using a study flier and email. The flyer was posted in all study ED sites and contained a link and QR code to the REDCap survey. The recruitment email was sent to all ED providers, nurses, and administrators/leaders and contained the REDCap survey link. Eligible individuals could view the study consent document and progress to the survey items. Consent was indicated by survey completion.

#### Measures

The context was assessed using the context scale of the Organizational Readiness to Change Assessment (ORCA) [40]. Prior research indicates the ORCA is appropriate for cross-sectional evaluation of organizational differences relevant to health intervention implementation [26, 41–43]. As our goal was to assess the ED context of the ORCA’s three scales, we used only the context scale, which consists of six subscales. Two subscales measure aspects of organizational culture (leadership culture and staff culture), one subscale assesses leadership practices, one assesses measurement, one assesses opinion leaders’ readiness to change, and one assesses resources to support practice changes in general [40]. The psychometric properties of the composite context scale (Cronbach alpha:0.85) and subscales (Cronbach alpha: 0.86–0.93) are strong.^20^ Demographic questions included items for sex, gender, race, and ethnicity. The final survey item asked about willingness to be contacted with questions and to participate in a future interview.

#### Interviews

Survey participants who indicated a willingness to be interviewed were contacted to schedule interviews. As only few survey respondents agreed to schedule the interview, a flier was also distributed to recruit additional interview participants. Interviews were conducted by a study team member (DS) using Zoom videoconferencing software and an interview guide informed by the Consolidated Framework for Implementation Research (CFIR) [44, 45]. Before the interview, participants were provided with an information sheet with details surrounding the GUIDED-HF intervention. Consent was indicated by participation in the interview and was also confirmed at the start of each interview.

### Data analysis

#### Quantitative analysis

A composite context score was calculated as the mean score across all 23 sub-scale items. Scores for each context subscale were calculated similarly. Missing responses were imputed if the number of non-missing responses exceeded 85% of the number of questions in the survey. Determining whether to impute data was made based on the full scale and not the individual subscales. Data were imputed using the full scale and applied to calculations of the overall scale and the subscales. Missing responses were imputed as the mean of the non-missing responses within a given person. Descriptive statistics are reported by healthcare system and by profession (i.e., leader, provider, nurse). Wilcoxon rank-sum tests and Kruskal–Wallis tests were used to assess differences across healthcare systems and professions, respectively. Results were analyzed using R statistical software (version 4.4.0).

#### Qualitative data

Interview data were analyzed using inductive rapid qualitative analysis [46]. This approach was used for practical and efficiency reasons and allowed for timely results during the pre-implementation period that would inform GUIDED-HF implementation in the real-world ED setting. The validity of the qualitative data was ensured by reviewing and verifying transcripts for accuracy prior to coding. Two research team members (DPS & ABS) together reviewed the transcripts and summarized each participant’s words as statements in a rapid analysis template in Excel (Additional File A). Participant summary statements were then combined in an Excel sheet, summarizing all participants’ statements by CFIR construct and healthcare system. Finally, the research team members identified contextual similarities and differences between the two healthcare systems.

#### Convergence of data sources

Once both sources of data were analyzed, these data were combined using a joint display, offering a side-by-side comparison of the quantitative and qualitative data. As highlighted in (Table 6), similar to prior work, [47] the CFIR constructs and associated qualitative summary statements were organized by ORCA domains to gain an understanding of the organizational context at each site. The joint display allowed the study team to compare the qualitative and quantitative data elements, see patterns of contextual similarities and differences between the two healthcare systems, and subsequently use these insights to refine the implementation strategies for each site.

## Results

### Sample demographic characteristics

The final survey sample consisted of 78 participants, with 35 (45%) from HC-PW and 43 (55%) from HC-MW (Table 2). The sample was comprised of providers (55%) and nurses (29%), with leaders making up the remainder (15%) (Table 3). Participants were predominately white (76%) with median age 37.0 (32.0, 41.0). Comparing participants across healthcare systems, HC-PW had more female (51% vs. 47%) and fewer white (71% vs. 79%) participants, compared with HC-MW (Table 2). A total of 14 participants completed interviews with an equal number from HC-MW and HC-PW agreeing to be interviewed. Of the 78 who completed the Context Assessment, 24 participants indicated an interest in being interviewed – 16 providers, 6 nurses, and 2 leaders. –Upon reaching out to confirm their willingness and schedule the interview, 11 subsequently scheduled and completed interviews (nine providers, one nurse, and two leaders). Subsequent recruitment using a study flyer resulted in four additional responses (3 providers and one nurse) and of these, two providers and one nurse completed the interviews. Due to scheduling difficulties or participant preferences, both individual and group interviews were conducted for a total of three group sessions (ranging from 2 to 6 individuals per group) and three individual interviews. Individual interviews occurred when scheduling difficulties prevented group interviews. No new themes emerged at the end these interviews, resulting in data saturation, and thus recruitment of additional interview participants was discontinued.

**Table 2:** Descriptive statistics of demographic data for the overall sample

	***N***	**HC-MW *N* = 43**	**HC-PW *N* = 35**	**Combined *N* = 78**
**Age**	69	38 [35, 43] (38.4 ± 9.2)	33 [31, 37.75] (35.6 ± 6.8)	37 [32, 41] (37.3 ± 8.5)
**Sex:**				
Male	78	49% (21)	49% (17)	49% (38)
Female		47% (20)	51% (18)	49% (38)
Prefer not to reply		5% (2)	0% (0)	3% (2)
**Race:**				
Asian	78	2% (1)	9% (3)	5% (4)
Black/African American		2% (1)	0% (0)	1% (1)
White		79% (34)	71% (25)	76% (59)
Multiple races		0% (0)	6% (2)	3% (2)
Prefer not to reply		16% (7)	14% (5)	15% (12)
Missing		0% (0)	0% (0)	0% (0)
**Survey**	78	51% (22)	49% (17)	50% (39)
**Profession:**				
Leader	78	14% (6)	17% (6)	15% (12)
Provider		60% (26)	49% (17)	55% (43)
Nurse		26% (11)	34% (12)	29% (23)

For continuous variables, *b* is the median [*a* is the 25th percentile, *c* is the 75th percentile], followed by the mean and standard deviation in parentheses. *N* is the number of non-missing values. Numbers after proportions are frequencies

*HC-MW* Healthcare system site in the Midwest, *HC-PW* Healthcare system site in the Pacific West

**Table 3:** Descriptive statistics of demographic data of GUIDED-HF implementation by profession

	***N***	**Leader**	**Provider**	**Nurse**	**Combined**
		***N* = 12**	***N* = 43**	***N* = 23**	***N* = 78**
**Age**	69	42 [39.5, 45.5] (42.4 ± 4.1)	35 [32, 38.5] 35.5 ± 4.9)	35.5 [30, 41.75] (36.8 ± 10.6)	37 [32, 41] (37.0 ± 7.4)
**Sex:**					
Male	78	50% (6)	58% (25)	30% (7)	49% (38)
Female		42% (5)	40% (17)	70% (16)	49% (38)
Prefer not to reply		8% (1)	2% (1)	0% (0)	3% (2)
**Race:**					
Asian	78	0% (0)	7% (3)	4% (1)	5% (4)
Black/African American		0% (0)	0% (0)	4% (1)	1% (1)
White		67% (8)	77% (33)	78% (18)	76% (59)
Multiple races		8% (1)	2% (1)	0% (0)	3% (2)
Prefer not to reply		25% (3)	14% (6)	13% (3)	15% (12)
Missing		0% (0)	0% (0)	0% (0)	0% (0)
**Survey**	78	33% (4)	47% (20)	65% (15)	50% (39)

For continuous variables, *b* is the median [*a* is the 25th percentile, *c* is the 75th percentile], followed by the mean and standard deviation in parentheses. *N* is the number of non-missing values. Numbers after proportions are frequencies

*HC-MW *Healthcare system site in the Midwest, *HC-PW *Healthcare system site in the Pacific West

### Convergence of findings: contextual assessment results and effects on implementation

Convergence of the quantitative and qualitative data informed the selection and refinement of implementation strategies for each site. The research team started with an initial list of strategies (Table 1), selected based on their prior experience with implementing interventions in ED and hospital settings. Context scale scores are reported by site (Table 4) and by profession (Table 5).

**Table 4:** Test of association for bsurvey results by ED location

	***N***	**HC-MW** ***N* = 43** **(*a b c*)**	**HC-PW** ***N* = 35** **(*a b c*)**	**Combined** ***N* = 78** **(*a b c*)**	***P*-value**
**Organizational Readiness to Change Assessment Context Scale, Overall**^a^	78	3.7 (3.4, 4.0)	3.7 (3.2, 4.0)	3.7 (3.4, 4.0)	0.29
Leadership culture	78	4.0 (3.3, 4.0)	4.0 (3.3, 4.0)	4.0 (3.3, 4.0)	0.69
Staff culture	78	4.0 (3.6, 4.2)	4.0 (3.5, 4.0)	4.0 (3.5, 4.2)	0.26
Leadership practice	78	4.0 (3.5, 4.2)	4.0 (3.2, 4.1)	4.0 (3.5, 4.2)	0.73
Measurement	78	4.0 (3.4, 4.0)	3.8 (3.0, 4.0)	3.8 (3.1, 4.0)	0.12
Readiness	78	4.0 (3.8, 4.0)	4.0 (4.0, 4.2)	4.0 (3.8, 4.2)	0.43
Resources	78	3.0 (2.5, 3.8)	3.0 (2.4, 3.5)	3.0 (2.5, 3.5)	0.26

For continuous variables, *b* is the median, (*a* is the 25th percentile, and *c* is the 75th percentile). *N* is the number of non-missing values

*ED* Emergency department, *HC-MW* Healthcare system site in the Midwest, *HC-PW* Healthcare system site in the Pacific West

Test used: Wilcoxon test

^a^Likert scale: 1 = *strongly disagree* to 5 = *strongly agree*

**Table 5:** Test of association for survey results by profession

	***N***	**Leader *N* = 12** **(*a b c*)**	**Provider *N* = 43** **(*a b c*)**	**Nurse *N* = 23** **(*a b c*)**	**Combined *N* = 78** **(*a b c*)**	***P*-value**
**Organizational Readiness to Change Assessment Context Scale, Overall**^a^	78	3.7 (3.5 4.0)	3.9 (3.5, 4.0)	3.6 (3.0, 3.9)	3.7 (3.4, 4.0)	**0.048**
Leadership culture	78	3.7 (3.7, 4.0)	4.0 (3.5, 4.3)	3.3 (2.8, 4.0)	4.0 (3.3, 4.0)	0.055
Staff culture	78	4.0 (3.8, 4.5)	4.0 (3.8, 4.2)	3.8 (3.5, 4.0)	4.0 (3.5, 4.2)	0.18
Leadership practice	78	3.9 (3.5, 4.2)	4.0 (3.5, 4.2)	3.8 (3.0, 4.0)	4.0 (3.5, 4.2)	0.13
Measurement	78	3.5 (3.0, 4.0)	4.0 (3.6, 4.0)	3.5 (3.0, 4.0)	3.8 (3.1, 4.0)	0.1
Readiness	78	4.0 (4.0, 4.1)	4.0 (3.8, 4.2)	3.5 4.0 4.1	3.8 4.0 4.2	0.72
Resources	78	3.0 (2.5, 3.6)	3.2 (2.8, 3.8)	2.0 2.8 3.1	2.5 3.0 3.5	**0.026**

For continuous variables, *b* is the median, *(a* is the 25th percentile, and *c* is the 75th percentile). *N* is the number of non-missing values

Test used: Kruskal–Wallis test

^a^Likert scale: 1 = *strongly disagree* to 5 = *strongly agree*

Convergence of the reported context scores (ORCA measures) and qualitative data (CFIR constructs) are reported in Table 6 and discussed next. CFIR constructs used in the interview guide and additional constructs identified were mapped to the mixed-methods data (see Additional File B).

**Table 6:** Convergence of quantitative and qualitative data

**ORCA Domain**^a^ and definition	**CFIR Domain**	**HC-MW** **Median (IQR)**	**HC-PW** **Median (IQR)**
**Organizational Readiness to Change Assessment Context Scale, overall** Quality of the overall organizational context for the program		3.7 (3.4, 4.0)	3.7 (3.2, 4.0)
**Organizational Culture**			
**Staff culture** Staff’s cooperativeness, receptiveness to change/innovation, and sense of personal responsibility for the quality of patient care	**Inner Setting:** compatibility; relative priority	4.0 (3.6, 4.2)	4.0 (3.5, 4.0)
		High staff turnover; positive interdepartmental relationships; high percentage of travel nurses; GUIDED-HF fit with values and clinical importance in ED setting	Core group of ED nurses identified as those who “get things done” regarding buy-in for new programs; lack of understanding of financial impact of ED recidivism; GUIDED-HF seen as adding to standard therapy and potential to reduce ED recidivism
**Leadership culture** Leadership rewards clinical innovation, solicits opinions of staff, and seeks ways to improve patient education and patient participation in care	**Inner Setting:** leadership engagement; e; incentives & rewards; networks & communication	4.0 (3.3, 4.0)	4.0 (3.3, 4.0)
		Distrust in leadership support for practice change; leadership perceived as will strongly endorse GUIDED-HF	Mismatch between leadership and staff values (financial vs. clinical impact); general sense of support; leadership perceived as will be supportive of GUIDED-HF
**Leadership Practice**			
**Formal leadership** Level to which formal leadership promotes teambuilding and communication, effective management of patient care improvement, and ability to clearly define responsibility areas for management and staff	**Inner Setting:** networks & communications; leadership engagement	4.0 (3.5, 4.2)	4.0 (3.2, 4.1)
		Providers identified leadership support as present for GHF specifically but feel unsupported for significant changes overall; part-time nursing administrators identified as critical for past implementation successes and RN education	Providers identified leadership support as present for GUIDED-HF; identified mismatch between what leadership cares about (financial impact) and what frontline clinicians care about (clinical outcomes)
**Measurement**			
**Measurement (leadership feedback)** Evaluation in terms of setting goals, tracking, and communicating performance	**Inner Setting:** goals & feedback; reflecting & evaluating	4.00 (3.4, 4.0)	3.8 (3.0, 4.0)
		Clinical performance feedback is anecdotal and not formal; time in the department is tracked, but data are not used for compensation	Minimal clinical metric feedback and mostly standard ED efficiency metrics (e.g., length of stay); Desire more positive outcomes feedback and clinically oriented metrics rather than throughput metrics
**Readiness to Change**			
**Opinion leaders’ readiness to change** A function of attitudes of opinion leaders for practice change in general	**Inner Setting:** readiness for implementation; opinion leaders; relative priority	4.0 (3.8, 4.0)	4.0 (4.0, 4.2)
		Very similar programs in place currently for other diseases; currently have a heart failure clinic for patient follow-up, but it is only available to patients who have a standing primary provider	Management of chronic illness is not a part of ED provider “mindset”; reports of challenging adoption of prior related programs because of provider resistance; no current competing initiatives
**Resources**			
**Resources** **context** Resources to support practice changes in general, once they have been made an organizational priority	**Inner Setting:** available resources; networks & communication; access to knowledge & information	3.0 (2.5, 3.8)	3.0 (2.4, 3.5)
		Resource availability is slim; existing infrastructure for health coaches; concern for time spent enrolling patients in the ED and will disrupt ED throughput metrics; lack of resources limited sustainability of other programs leading to increased provider time/clinical workload commitment	Providers sometimes questioned whether sufficient resources are available for sustainability, especially within underserved populations; space and electronic barriers to having a research coordinator in the ED; bed shortages and overcrowding not allowing enough time for enrollment in the ED; poor workflow infrastructure for setting up follow-up care

Note. Qualitative data reflect summary statements across all participants for each site

^a^Likert scale: 1 = *strongly disagree* to 5 = *strongly agree*

The context scale assesses the quality of an organization’s context to support practice change. On a scale of 1 = *strongly disagree* to 5 = *strongly agree*, we observed a moderate composite context score (3.7 [3.4, 4.0]). Scores were similar across the two healthcare systems and aligned with interview findings demonstrating the participants’ overall optimism for incorporating GUIDED-HF as a practice change. Composite context scores differed across professions (*p* = 0.048), with providers reporting higher scores (3.9 [3.5, 4.0]) than leaders (3.7 [3.5, 4.0]) or nurses (3.6 [3.0, 3.9]). (See Tables 4 and 5). Post hoc pairwise tests using Wilcoxon Rank Sum tests showed that the significant difference was driven by the difference between Providers and Nurses (difference in median: 0.3; Bonferroni-corrected *p*-value: 0.04), and that there is no difference between Leader and Provider (difference in median: −0.2; Bonferroni-corrected *p*-value: 1.0), and between Leader and Nurse (difference in median: 0.1; Bonferroni-corrected *p*-value: 0.66).

### Organizational culture: two dimensions

Leadership and staff culture subscale scores were similar (leadership culture: 4.0 [3.3, 4.0]; staff culture: 4.0 [3.5, 4.2]), indicating moderately high organizational culture for change. Leadership culture scores at HC-MW and HC-PW were also similar (4.0 [3.3, 4.0]), as were staff culture scores (HC-MW: 4.0 [3.6, 4.2]; HC-PW: 4.0 [3.5, 4.0]) (Table 3). However, salient differences arose within the qualitative interviews. HC-PW experienced significant staff turnover, especially among nursing staff, whereas HC-MW identified a stable “core group” of ED nurses known for leading practice change among other ED staff and embodying quality care.

#### Implementation based on organizational culture findings

Organizational culture differences were observed for educational session frequency, who should serve as site champions, and who should be part of consensus discussions. At HC-PW, the research team targeted dedicated ED faculty as champions, as they were more likely to be consistently present and employed by the unit throughout the study period. In contrast, HC-MW’s high-performing and respected “core group” of ED nurses were identified as pivotal to serving as site champions. High nursing staff turnover at HC-PW also signaled the need to repeat HC-PW’s educational sessions at least every 4–6 months to account for travel nursing contracts and staff turnover (Table 7).

**Table 7:** Implementation strategies and tailoring by site

A. CATEGORY: Planning		
	**HC-MW**	**HC-PW**
**A.1. Develop stakeholder buy-in**		
**-Use of champions**	ED staff, RNs (especially nursing administrators), department heads, physicians as champions	Dedicated ED faculty as champions
**-Consensus discussions**	Include experienced **nurses**; highlight GUIDED-HF’s alignment with larger HF readmission reduction initiative	Include medical director, **nursing** leadership, specialists, primary care; focus on understanding potential sources of provider resistance; in-person only
**A.2. Marketing**	Use visuals; use study fliers; **workstation prompts**; newsletter piece	At ED faculty and operations meetings; put executive summary in EPIC; **workstation prompts**; newsletter, and swag with study logo
**B. CATEGORY: Educate**		
**B.1. Conduct educational meetings**	Use “train the trainer” model; host during **staff meetings** and nurse huddles	Repeat educational meetings every 4–6 months because of staff turnover; host during **ED faculty and operations meetings**; include ancillary staff in education
**B.2. Develop and distribute educational material**	Use in-person, not email/printed materials/computer modules	Use in-person and email distribution strategy
**C. CATEGORY: Quality Management**		
**Adapting workflow processes**	**Use BPA**; design BPA with “opt-in” approach	**Use BPA**; design BPA with “opt-out” approach
**D. CATEGORY: Restructuring**		
**Audit and feedback**	**Provide feedback data in aggregate** and at the individual level	Focus on positive rather than negative feedback and clinical outcomes; **provide data in aggregate** for ED providers

Bold indicates similarities between the two sites

### Leadership practice

The formal leadership subscale score **(**4.0 [3.5, 4.2]) demonstrated moderate leadership practice for implementing GUIDED-HF. Qualitative findings suggested leadership support for GUIDED-HF workflow development was essential for program success. In both healthcare systems, the involvement and support of the ED medical director and ED nurses were highlighted as important**.** Both sites indicated believing their respective leadership teams supported incorporating GUIDED-HF specifically and cultivated moderately strong leadership contexts **(**HC-MW: 4.0 [3.5, 4.2]; HC-PW: 4.0 [3.2, 4.1]). But, drawing on the qualitative findings, sites appeared uncertain regarding leadership’s follow through because of site-specific concerns. Whereas a mismatch between leadership and staff values (i.e., financial impact vs. clinical outcomes) contributed to hesitation among HC-PW clinicians, a sense of general lack of support for significant practice change defined HC-MW clinicians’ hesitation. HC-MW’s global distrust regarding leadership’s practice change support alerted the research team to the importance of addressing this distrust during implementation planning.

The score on the measurement context subscale, which assesses leadership setting goals, information sharing, and feedback, was moderately high (3.8 [3.1, 4.0]). The context score for measurement scored higher at HC-MW (4.0, [3.4, 4.0]) compared to HC-PW (3.8 [3.0, 4.0]). HC-PW clinicians expressed concern over the use of ED throughput metrics such as length of stay and time to be seen rather than metrics specifically related to clinical outcomes. They also perceived concern for throughput metrics occasionally being used punitively. At HC-MW, clinicians noted metrics were generally not used in compensation/performance decisions. These qualitative insights explain conflicting results in the quantitative data; specifically, HC-PW participants reported lower measurement context scores, though leadership is perceived as using “more” measurement overall. HC-PW clinicians preferred to receive performance feedback only in aggregate at the group level rather than individually. HC-MW clinicians appeared open to both individual- and group-level feedback. These insights were invaluable when considering how to tailor audit and feedback and adaptation of workflow processes.

#### Implementation based on leadership practice findings

The research team dedicated resources to show recognition and support for clinician and nurse participation at both sites. We worked with local leaders to provide site specific recognition. The research team also created a process to identify clinicians with low GUIDED-HF adoption rates and took steps to encourage future adoption and patient referral. Audit and feedback processes were planned to reflect the site preferences noted above. Survey and interview insights stressed the importance of designing clinically relevant proxies for clinician performance. For example, rather than providing clinicians with feedback regarding potential ED cost savings through GUIDED-HF referral, the study team focused subsequent updates on clinically oriented metrics such as changes in ED readmissions and ED follow-up visits. Feedback for both sites was structured using positive framing (Table 7).

### Opinion leaders’ readiness to change

Although the ORCA readiness context subscale score (4.0 [3.8, 4.2]) suggested strong readiness for GUIDED-HF and practice change overall within the two healthcare systems (HC-MW: 4.0 [3.8, 4.0]; HC-PW: 4.0 [4.0, 4.2]), qualitative findings helped identify critical insights to further improve implementation readiness. Clinicians and staff at HC-PW reported a history of lackluster adoption of prior program initiatives due especially to provider resistance. HC-MW staff and clinicians suggested their ED already incorporates programs like GUIDED-HF for other clinical conditions and noted the system at large is currently engaged in an HF readmission reduction initiative.

#### Implementation based on readiness for change findings

These insights informed the deployment of GUIDED-HF and its marketing to clinicians. Pre-implementation marketing and education included informing providers of GUIDED-HF and the importance of their responding to the best practice alert** (**BPA) and referring patients as applicable. The unique role of the self-care coach in contacting patients and executing the intervention was emphasized, as was the transfer of workload from providers to self-care coaches. Educational materials and content were developed to emphasize the benefits of GUIDED-HF participation for patients. Identifying GUIDED-HF’s similarities to other positively viewed programs was key, and alignment with HC-MW’s larger HF readmission initiative was stressed in marketing materials and consensus discussions (Table 7).

### Resources

The resources context subscale had the lowest overall score (3.0 [2.5, 3.5]). No differences were observed in scores between the two healthcare systems (Table 3). However, a statistically significant difference was observed among professions with a higher scored observed for providers for the availability of necessary resources to support practice change (3.2 [2.8, 3.8]) followed by nurses (2.8 [2.0, 3.1]) and leaders (3.0 [2.5, 3.6]) (*p* = 0.026). Qualitative interview findings showed that, at HC-PW, participants were concerned about the lack of resources, space, and bed shortages to support GUIDED-HF’s ongoing sustainability. HC-MW participants shared similar concerns and noted a need for infrastructure to support self-care coaches, with no significant infrastructure changes necessary to implement GUIDED-HF. Despite resource concerns, they displayed overwhelming optimism for GUIDED-HF and perceived GUIDED-HF as innovative and highly compatible with their existing practices in the ED. Participants remarked on the advantage of GUIDED-HF over other alternatives for discharging patients with AHF. They believed GUIDED-HF would reduce provider workloads and help overcome patient barriers to seeking care (particularly patient-related literacy challenges).

Interviewed participants reported multiple possible solutions to alleviate resource concerns. Participants reported using a BPA to trigger referral should be vetted and resource allocation should focus on equipping self-care coaches specifically to execute GUIDED-HF. Participants noted the need to train ED staff on GUIDED-HF using different training modalities and educational strategies. The message communicating the importance of implementing GUIDED-HF needed to be clear, simple, and standardized. Finally, feedback was seen as an essential component of engaging ED staff members. Participants mentioned specific needs when providing feedback, including longitudinal data on patient outcomes (e.g., patient follow-up visits and hospitalizations). Sites differed on the content, type, mode, and frequency of feedback they wanted.

#### Implementation based on resources findings

To address these concerns, we emphasized the GUIDED-HF intervention structure during education and marketing. Providers were reminded of the BPA trigger as the only GUIDED-HF component occurring in the ED setting, with self-care coach follow-up after ED discharge. This structure limited the use of ED resources, including provider time and space. GUIDED-HF’s role of ensuring follow-up care of patients with AHF after ED discharge was also emphasized during training events (Table 7).

## Discussion

Despite a robust commitment to developing clinical practice guidelines and scientific reviews to keep pace with the rapid advancements in evidence-based emergency medicine, significant knowledge-to-practice gaps and care variation remain [48]. We utilized implementation science strategies to reduce this evidence-to-practice gap by presenting a novel, theory-informed example of organizing and understanding quantitative and qualitative contextual findings. Our goal was to identify implementation determinants in diverse ED settings to inform the development of implementation strategies. By developing, specifying, and reporting effective implementation strategies and mapping these strategies to the contextual settings where they will be most effective, our approach allows EDs to select the implementation strategies most suited to the determinants specific to their context. This work aligns with existing recommendations [35] and provides a starting point for clinicians and implementers as they develop implementation strategies that address their local ED setting.

Although the fast-paced environment of the ED, with high patient complexity and staff turnover is unquestionably unique, the current and prior studies’ results suggest ED settings experience similar barriers to practice change and needs for refined implementation strategies as other care specialties. In this study, ED sites identified insufficient resources and education for practice change as major barriers. This finding was consistent with a previous scoping review of determinants of prediction models in ED settings that found limited resources and education as significant determinants of successful implementation [49]. These needs also align with other care specialties and thus suggest that tailored implementation strategies developed in other care contexts (e.g., the ERIC taxonomy [20]) might be useful in emergency care settings.

This mixed-methods convergent analytic approach improves multiple aspects of implementation planning and provides opportunities for future implementation science research. Rather than using only quantitative or qualitative data to understand the implementation context, this study used complimentary methods, that is integrating quantitative and qualitative data, to gain a comprehensive understanding of contextual determinants. Minimal differences between sites were observed in the quantitative data, with the exception of the measurement sub-scale with questions that focused on the sharing of performance measures and feedback. The qualitative data elucidated reasons for the different ratings of this sub-scale between the two sites. Overall, the quantitative ORCA context scale allowed for a quick and high-level evaluation of site contextual factors as determinants of implementation But, the qualitative data brought to light the nuanced differences between the two healthcare systems and provided greater insight into specific site differences and implementation preferences that underscored the need for a tailored approach to address the specific concerns and preferences of the local context.. The convergence of the qualitative and quantitative findings was imperative for understanding how contextual challenges uniquely manifested in each system, allowing us to develop a cohesive implementation approach. We suggest prospective mixed-methods assessment is key to understanding ED contextual determinants of implementation.

The insights gained from this study can inform the development of tailored strategies to enhance the implementation of similarly complex interventions in ED settings. For instance, at sites like HC-PW, research teams in future may need to focus on addressing provider resistance and building trust, while at sites like HC-MW, they can build upon the existing experience and organizational focus on heart failure to facilitate a more seamless integration of the GUIDED-HF program. The findings also underscore the importance of assessing and addressing the multifaceted aspects of organizational context and culture when implementing practice changes in healthcare settings. For example, the moderate composite context score and moderately high organizational culture provide a generally supportive environment, but the observed differences across professions and healthcare systems highlight the need for a tailored approach that considers the unique characteristics and dynamics of each organization. The moderate leadership practice score and moderately high measurement context score suggest a generally supportive environment, but the site-specific differences identified through the qualitative insights further highlight the need for a tailored approach that addresses the unique characteristics and dynamics of each organization. Our work adds to prior recommendations for implementation in EDs, stressing the importance of understanding implementation barriers and facilitators, identifying strong local champions, having a study start-up meeting with local stakeholders, regularly communicating with each site, and using incentives to encourage compliance. Our work similarly demonstrates the need for consensus discussions with ED stakeholders and the use of champions and incentives to support staff implementing interventions in real-world settings. We also demonstrated that, with some site-specific adaptation, similar implementation strategies could be deployed across two diverse healthcare systems and their EDs. For example, champions were used at all sites, but the selection of who these would be was determined by site preference.

Some limitations of this study should be noted. As the GUIDED-HF implementation-effectiveness study is ongoing, this article details implementation determinants and the selection and refinement of implementation strategies but does not report on the outcomes of these implementation strategies in ED settings. It is unclear how our selected and refined strategies will impact implementation outcomes of interest (e.g., reach, adoption, and maintenance). But, our ongoing hybrid type II implementation formative evaluation will help determine the impact of these strategies. Additionally, stakeholder groups (e.g., staff, nurses, leaders, physicians) were not equally represented across sites. However, prior work suggests over- or under-representation of stakeholder groups is unlikely to impact the identification of relevant determinants or strategies for successful evidence-based practice implementation [50]. Also, because of the small sample of the different professional groups, comparing the qualitative findings across the various small groups was not possible and should be explored in future research.

Overall, our experience suggests converging quantitative and qualitative data is a low-burden, efficient, and beneficial approach for research teams desiring to prospectively refine implementation strategies. Our novel process can serve as a foundation for future implementation research work in the ED setting.

## Conclusion

This study identified crucial contextual determinants to consider when selecting and refining implementation strategies in ED settings. Special consideration to the multifaceted aspects of the ED and broader organizational context and culture is crucial to facilitate practice change for effective implementation. This study provides ED settings with guidance for future implementation by mapping implementation strategies to contextual determinants, thereby allowing other ED settings to determine the strategies that might be most effective in their particular setting.

## Supplementary Information

Supplementary Material 1: Additional file A: Rapid Qualitative Matrix Template

Supplementary Material 2: Additional file B: CFIR constructs mapped to interview questions

## Data Availability

The quantitative and qualitative data supporting the conclusions of this article will not be publicly available due to ongoing data analyses related to all study aims.
