# Supplementary material for: A mixed-methods observational study of strategies for success in implementation science: overcoming emergency departments hurdles
Source: BMC Health Serv Res. 2025 Jan 27;25:147. doi: 10.1186/s12913-024-12102-9 (PMC11770910; doi:10.1186/s12913-024-12102-9)
Supplement: Supplementary file 1 — Supplementary Material 1: Additional file A: Rapid Qualitative Matrix Template [file 12913_2024_12102_MOESM1_ESM.docx]

**A Mixed-Methods Observational Study of Strategies for Success in Implementation Science: Overcoming Emergency Department Hurdles**

**ADDITIONAL FILE A**

| **Rapid Qualitative Matrix Template** | | | |
| --- | --- | --- | --- |
| **Interview 1** | **Interview 2** | **SUMMARY STATEMENTS** | **BARRIERS AND FACILITATORS** |
| **Hosp A** | **Hosp A** | **Hosp A** | **Hosp A** |
|  |  |  |  |
|  |  |  |  |
|  |  |  |  |
|  |  |  |  |
| **Interview 1** | **Interview 2** | **SUMMARY STATEMENTS** | **BARRIERS AND FACILITATORS** |
| **Hosp B** | **Hosp B** | **Hosp B** | **Hosp B** |
|  |  |  |  |
|  |  |  |  |
|  |  |  |  |
|  |  |  |  |
|  |  |  |  |
|  |  |  |  |
|  |  |  |  |
