# Supplementary material for: A mixed-methods observational study of strategies for success in implementation science: overcoming emergency departments hurdles
Source: BMC Health Serv Res. 2025 Jan 27;25:147. doi: 10.1186/s12913-024-12102-9 (PMC11770910; doi:10.1186/s12913-024-12102-9)
Supplement: Supplementary file 2 — Supplementary Material 2: Additional file B: CFIR constructs mapped to interview questions [file 12913_2024_12102_MOESM2_ESM.docx]

**A Mixed-Methods Observational Study of Strategies for Success in Implementation Science: Overcoming Emergency Department Hurdles**

**ADDITIONAL FILE B^*^**

| **CFIR CONSTRUCTS MAPPED TO INTERVIEW QUESTIONS** | | |  |
| --- | --- | --- | --- |
| **CFIR CONSTRUCT** | **Concept within CFIR Category** | **CIFR guided interview questions (examples)** |  |
| **INNER SETTING** |  |  |  |
|  | **Compatibility** | How well does GUIDED-HF fit with your values and norms [ for patient care] and the values and norms within the organization? |  |
|  | **Relative Priority** | What kinds of high-priority initiatives or activities are already happening in your setting for discharged patients?   1. How might these priorities affect the implementation of GUIDED-HF? 2. How can we learn from them to ensure GUIDED-HF success? |  |
|  | **Goals & Feedback** | How does implementation of GUIDED-HF align with [ED or organizational] goals related to caring for patients with a primary diagnosis of AHF discharged from the ED? |  |
|  | **Leadership Engagement** | What kind of support or actions could we expect from leaders in your organization (both in and outside the ED) to help make implementation successful?   - - Who are these leaders?   - What types of barriers might they assume or create? |  |
|  | **Available Resources** | When implementing interventions for patient care improvement, do you usually have sufficient resources for it?   - - [If Yes] What resources can you usually count on? Are there any other resources that you would have liked to receive?   - What resources will be easy to procure?   - [If no] What resources will not be available? |  |
|  | **Networks & Communications** | When you need to get something done or to solve a problem, who are your "go-to" people? |  |
|  | **Access to Knowledge & Information** | What kind of training should we provide on GUIDED-HF to support you to carry out the roles and responsibilities expected of you in the context of this work? Can you explain? |  |
|  | **Structural Characteristics** | 1. What kinds of infrastructure changes will be needed to accommodate GUIDED-HF? [*if participants have a problem answering, can prompt them with these examples:* social architecture, age, maturity, size, or physical layout]    - Changes in scope of practice? Changes in formal policies? Changes in information systems or electronic records systems? Other?   Can you describe the process that will be needed to make these changes? |  |
| **Characteristics of Individuals** |  |  |  |
|  | **Knowledge & Beliefs about GUIDED-HF** | 1. Do you think GUIDED-HF will be effective in your setting?    - Why or why not? |  |
|  | **Self-efficacy** | 1. How confident are you that you will be able to use GUIDED-HF?    - What gives you that level of confidence (or lack of confidence)? |  |
| **Process** |  |  |  |
| Planning | **Opinion Leaders** | Who are the key influential individuals we need to get buy-in from to implement GUIDED-HF effectively? |  |
|  | **Champions** | 1. Other than the formal implementation leader (if you have one), are there people in your organization who are likely to champion (go above and beyond what might be expected) GUIDED-HF?    - Were they formally appointed in this position, or was it an informal role?    - What position do these champions have in your organization?   How do you think they will help with implementation? Getting people to use GUIDED-HF? |  |
|  | **Engaging stakeholders** | 1. What should we use as our communication or education strategy (not including training, see Access to Knowledge and Information) for getting the word out about GUIDED-HF?   What materials/modes/venues should we use? For example, e-bulletin boards, emails, brochures? |  |
|  | **Reflecting & Evaluating** | 1. During the study, we will provide feedback reports about our implementation and outcomes associated with (?) GUIDED-HF.    - What should feedback look like? Content, mode, form?    - How often would you like to get them?    - How helpful do you think it would be?    - How should we share results of the evaluation to stakeholders? | |
| **GUIDED-HF**  **Characteristics** |  |  | |
|  | **Relative Advantage** | 1. How does GUIDED-HF compare to other alternatives for discharge patients with AHF that may have been considered or that you know about?    - What advantages does GUIDED-HF have compared to these other programs?    - What disadvantages does GUIDED-HF have compared to these other programs? | |
|  | **Adaptability** | 1. What kinds of changes or alterations do you think we will need to make to GUIDED-HF so it will work effectively in your setting? *Please explain* | |
| * Some interview questions from CFIR resource file ([www.cfirguide.org](http://www.cfirguide.org)) were adapted and used for the study. | | | |
